# An effective validation of analytical method for determination of a polar complexing agent: the illustrative case of cytotoxic bleomycin

**DOI:** 10.1007/s00216-023-04675-x

**Published:** 2023-04-12

**Authors:** Helena Plesnik, Masa Bosnjak, Maja Cemazar, Gregor Sersa, Tina Kosjek

**Affiliations:** 1 Department of Environmental Sciences, Jozef Stefan Institute, Jamova 39, 1000 Ljubljana, Slovenia; 2 International Postgraduate School Jozef Stefan, Jamova cesta 39, 1000 Ljubljana, Slovenia; 3 Department of Experimental Oncology, Institute of Oncology Ljubljana, Zaloska 2, 1000 Ljubljana, Slovenia; 4 Faculty of Pharmacy, University of Ljubljana, Askerceva 7, 1000 Ljubljana, Slovenia; 5 Faculty of Health Sciences, University of Primorska, Polje 42, 6310 Izola, Slovenia; 6 Faculty of Health Sciences, University of Ljubljana, Zdravstvena pot 5, 1000 Ljubljana, Slovenia

**Keywords:** Liquid chromatography, Mass spectrometry, Biological, Pharmaceutical, Measurement uncertainty, Stability

## Abstract

The effectiveness of highly polar agents in cancer treatment is well recognized, but their physicochemical properties make their analytical determination a demanding task. Their analysis requires peculiar sample preparation and chromatographic separation, which heavily impacts the precision of such an analytical method. As a case study, we chose a polar cytotoxic bleomycin, which is a mixture of complexing congeners with relatively high molecular mass, a fact that creates an added challenge in regard to its detection via electrospray mass spectrometry. These issues combined lead to a deprived method performance, so the aim of this study is manifold, i.e., to optimize, validate, and establish quality performance measures for determination of bleomycin in pharmaceutical and biological specimens. Quantification of bleomycin is done at diametrically different concentration levels: at the concentrations relevant for analysis of pharmaceutical dosage forms it is based on a direct reversed-phase HPLC-UV detection, involving minimum sample pretreatment. On the contrary, analysis of bleomycin in biological specimens requires phospholipid removal and protein precipitation followed by HILIC chromatography with MS/MS detection of bleomycin A2 and B2 copper complexes being the predominant species. This study further attempts to solve the traceability issue in the absence of certified reference standards, determines measurement uncertainty, investigates BLM stability and method performance characteristics, and, last but not least, provides an explanatory example of how a method quality assurance procedure should be established in case of an exceedingly complex analytical method.

**Graphical abstract:**

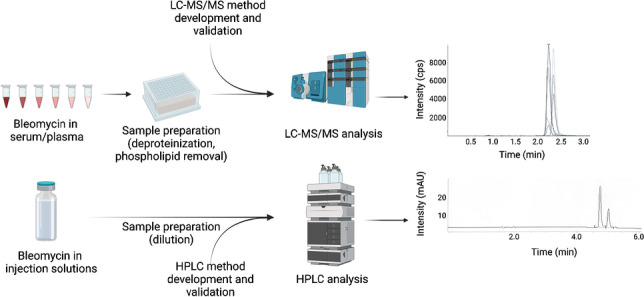

**Supplementary Information:**

The online version contains supplementary material available at 10.1007/s00216-023-04675-x.

## Introduction

Bleomycin (BLM) is a common term for a group of structurally related cytostatic antibiotics, isolated from *Streptomyces verticillus*. Due to its high cytotoxicity, it is used in the treatment of various neoplasms, particularly squamous carcinoma, lymphoma, and testicular carcinoma [1]. It was recently proven to also be effective against vascular malformations [2]. Although injected in the apo form, the cytotoxic activity is exhibited by its metal complexes or metallobleomycins. These generate reactive oxygen species and thereby oxidatively damage DNA, causing single- and double-strand breaks, ultimately destroying the cell [3]. The exact mechanism of action has however yet to be fully elucidated.

To achieve the desired cytotoxic effect in patients, a sufficient amount of bleomycin must reach its site of action, located inside the cell nuclei. The transition of bleomycin through cell membranes is however hindered due to its size and rather hydrophilic nature. Therefore, only a small portion of molecules is able to enter the cells. Simply increasing the administered dose to reach the desired effect does not present a viable solution as doing so would also amplify the toxic side effects, already observed at the currently used doses in a significant fraction of patients (with up to 46% experiencing significant morbidity) [4]. This problem is stimulating the development of new approaches, one promising example being electrochemotherapy (ECT), where electrical pulses are applied to transiently increase the permeability of cell membranes, thereby facilitating the passage of BLM into cells [5]. ECT is still being optimized but has already been proven to be effective in tumor as well as vascular malformations therapy [2, 6, 7]. Another obstacle to achieving sufficiently high concentrations of bleomycin at the site of its action is the presence of bleomycin hydrolase (BLMH), a cytoplasmic protease, causing deamination and thereby inactivation of bleomycin. Despite being tissue non-specific, this enzyme is overexpressed in some tumors and further reduces the amount of intact bleomycin reaching its site of action [8, 9].

Quantitative analyses of bleomycin for assay testing of its pharmaceutical form (injection solution powder) are, as pure and rather concentrated solutions can be prepared with the powder, routinely performed using simple methods based on HPLC separation with UV detection as described in the European Pharmacopoeia and require little to no pretreatment [10]. A different approach must be considered with clinical samples. Biological matrices such as blood or tissue are namely relatively complex, which implies extensive sample preparation. In addition, BLM is present at considerably lower concentrations in such samples, requiring the use of highly sensitive instruments. The newest published method, meeting such requirements, describes sample preparation employing solid-phase extraction and is based on UHPLC-MS/MS, and reaches a lower limit of quantification (LLOQ) in the ng/mL range [11].

**Fig. 1:**
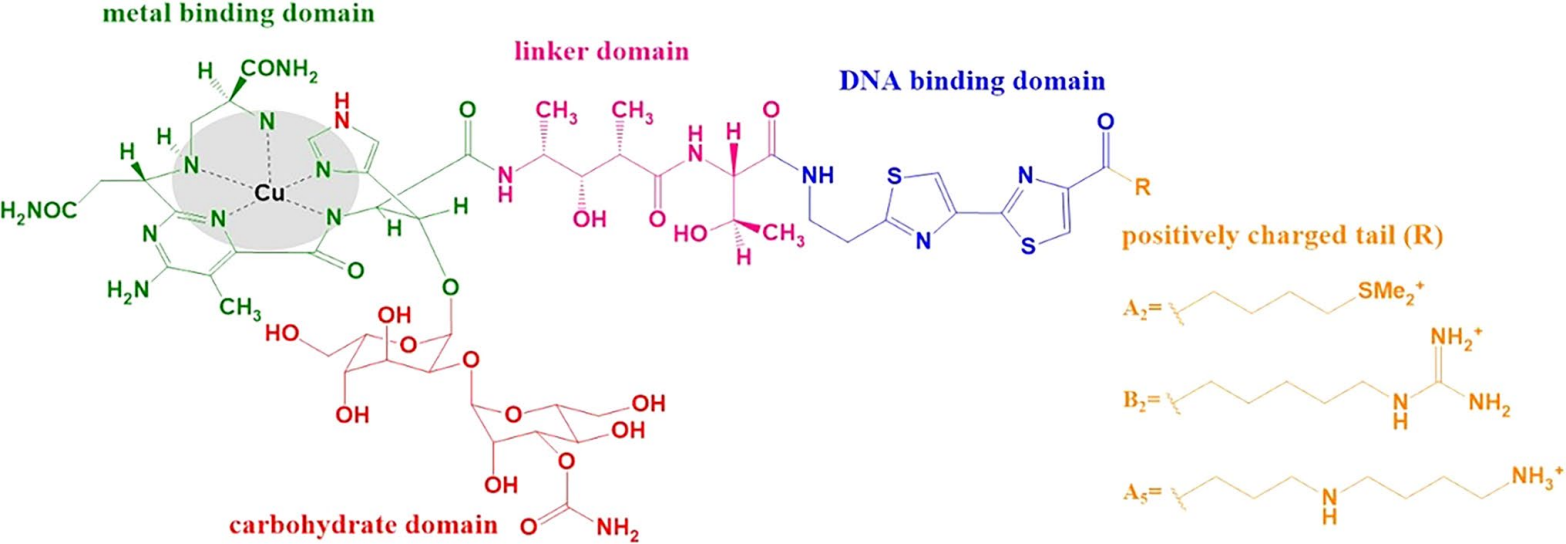
Structure of a BLM-Cu complex (adapted from [1])

High-quality analytical procedures are of critical importance for monitoring bleomycin concentrations in patients undergoing already established treatment regimes, but also support their further optimization or development of new therapeutic approaches. Developing a well-performing analytical method comes with various challenges, one of them being the heterogeneity of bleomycin, which is not a single molecule but rather a compost of multiple very similar fractions (A1–A6; B1–B5), only varying in the structure of the C-terminal substituent [12]. Its structure with main functional domains is shown in Fig. 1. Clinically administered bleomycin (Bleomycin Sulphate USP) largely consists of BLM-A2 (55–70%) and BLM-B2 (25–32%) fractions, but trace amounts of others may also be present [10, 12, 13].

Bleomycin’s tendency to form complexes with a range of redox-active metals (Fe, Cu, Ni, Mn, and Zn) plays an important role in its biological activity [1]. The highest binding affinity is shown towards copper ions, which bind in vivo as well as in vitro [11, 14]. Therefore, although administered in a metal-free form, BLM in the blood is present in the form of a copper complex. Complex formation needs to be accounted for in the case of mass spectrometric detection, as metallobleomycins differ from their corresponding native molecules in mass.

A limited number of analytical methods have been developed for the detection of bleomycin in biological samples. The early methods mainly utilized HPLC separation and identification techniques such as UV absorption, radioimmunoassay, and fluorescence detection, which demonstrate limited selectivity being unable to distinguish between BLM fractions [13, 15–17]. Striving towards better selectivity, some studies have been able to develop methods that managed to separate the predominant fractions of bleomycin (A2 and B2), using ion-paired reversed-phase HPLC [12, 13, 18, 19]. They however demonstrated either a relatively high detection limit [13] or a narrow linear concentration range [12]. After comparing the formerly often-used reversed-phase to hydrophilic interaction chromatography (HILIC), specifically designed for the analysis of polar compounds, Galba et al. [20] demonstrated the advantages of the latter. In addition to better retention, separation efficiency, and selectivity, the use of a HILIC column eliminates the need for adding ion-pairing reagents in the mobile phase. Similarly, our group resorted to a HILIC-like BEH Amide column, developing a method with high-resolution MS (HRMS) quantitative determination of the main bleomycin fractions that successfully separates their Cu(II) complexes [11]. Bleomycin’s high affinity towards copper ions and their presence in biological samples make the complex a sensible choice of analyte. Despite providing results with high selectivity, sensitivity, and low detection limits for both predominant bleomycin fractions, it relies on an HR mass analyzer, not readily available or widespread in the clinical setting. A method using more accessible instrumentation would therefore mean a great advantage, allowing its wider use.

The method for the quantitative determination of BLM in biological tissues was published in 2016 and was for the time being the only reliable choice for such analysis at clinical levels. Since then, new sorbent materials have been brought to the market, and along grew the need for simplification in terms of labor invested, time, and materials consumed, thus facilitating the analyses of sizeable batches of samples. This study reveals the results of analytical method optimization and its revalidation compliant with the European Medicines Agency (EMA) guidelines [21]. Furthermore, it attempts to solve the traceability issue, determines measurement uncertainty, investigates BLM stability and method performance characteristics, and, last but not least, this study sets an explanatory example of how method quality assurance procedure should be established in the case of an exceedingly complex analytical method.

## Experimental/materials and methods

*Caution:* BLM is cytotoxic, genotoxic, teratogenic, and mutagenic *and should be handled with care* [22, 23].

### Chemicals and materials

Primary reference standard: bleomycin sulfate salt (CAS No.: 9041-93-4) was purchased in a metal-free form with a declared purity of 95.7% (C55H84N17O21S3 × H2SO4) from Santa Cruz Biotechnology, Inc. (Dallas, TX). Cross-check reference standard bleomycin sulfate, also in metal-free form, was purchased with as declared purity of ≥95% from Cayman Chemical (Ann Arbor, MI). The pharmaceutical dosage form Bleomedac®, a powder solution for injection with the declared amount of bleomycin sulfate 15,000 IU that corresponds to the biological activity of 15 mg BLM, is produced by Medac GmbH (Wedel, Germany). Bleomycin A5 hydrochloride (C57H89N19O21S2 × HCl, CAS: 55658-47-4) used as an internal standard was purchased from LKT Laboratories, Inc. (St. Paul, MN) in a metal-free form and had a declared purity of ≥ 90%. The agent for control of metal complex formation was CuSO4 × 5H2O (Alkaloid Skopje, Macedonia). Mobile phase additives ammonium formate (≥99.0%) and formic acid (99%) were purchased at Sigma-Aldrich Corp. and Carlo Erba (Val de Reuil, France), respectively, both of LC-MS purity grade. Acetonitrile and water used as the mobile phases were of LC-MS purity, and all solvents used in sample prep (methanol, water, acetonitrile) were of analytical grade purity.

Normal human serum was purchased from Sigma-Aldrich (St. Louis, MO, USA). Blood plasma was acquired by using Vacutainer® heparin collection tubes.

The chromatographic columns used were Acquity UPLC BEH Amide column with dimensions 130 Å, 1.7 μm, 2.1 mm × 50 mm (Waters Corp., Milford, MA, USA) and Zorbax® Eclipse XDB-C18 column with dimensions 5 μm, 4.6 mm × 150 mm. For sample preparation, the following sorbent forms were used: Oasis HLB 96-well plates (30 mg sorbent per well, 30 μm), Oasis HLB 1cc (30 mg) extraction cartridges, and Ostro protein precipitation and phospholipid removal plates, 25 mg (Waters Corp.). Filtering of the samples before the analysis was performed on 0.2 µm Phenex^TM^ regenerated cellulose membrane syringe filters (Phenomenex, Torrance, CA).

The software used included ChemDraw 14.0 (Perkin-Elmer Corp., Norwalk, CT) and Analyst 1.6.3 (AB Sciex, Framingham, MA).

### Preparation of standard and working solutions

The stock solution of BLM was prepared by dissolving 10 mg of bleomycin sulfate primary reference standard in 10 mL of methanol and Milli-Q water mixture (8:2) and was kept at −18 °C. The stock solution of the cross-check reference standard was prepared the same way. The working standards at concentrations 0.1, 1, 5, and 10 μg/mL were freshly prepared before the analysis by diluting the BLM stock solution in 0.1% formic acid.

The internal standard (bleomycin A5) was prepared as a stock solution by dissolving 5 mg in 10 mL of methanol and Milli-Q water mixture (8:2) and was kept at −18 °C. The working standard was freshly prepared every time prior to analysis by diluting the stock solution to the concentration of 5 μg/mL.

The LC-MS method calibration and quality control standards were prepared by spiking the serum or plasma, their concentrations ranging from 6 to 1500 ng/mL. The HPLC method validation standards were prepared in concentrations 2.5–40 μg/mL BLM in Milli-Q water.

CuSO4, the agent for control of metal complex formation, was prepared by dissolving 1.22 mg of CuSO4·5H2O in 20 mL of Milli-Q water and further diluted to 3.05 μg/mL. It was later added into serum in a molar concentration that exceeded that of the analyte at the highest calibration point and internal standard combined.

NH4COO- measuring 200 mM was prepared by diluting 1.261 g in 100 mL of Milli-Q water. The aqueous mobile phase for the LC-MS method (10 mM ammonium formate with 0.1% formic acid) was prepared by 20 times diluting 200 mM ammonium formate and addition of 500 µL formic acid per 500 mL of buffer solution. The aqueous mobile phase for the HPLC method (20 mM ammonium formate with 0.1% formic acid) was prepared by 10 times diluting 200 mM NH4COOH and addition of 500 µL formic acid per 500 mL of buffer solution.

### Sample preparation

Two hundred microliters of either plasma or serum was put into 1.5-mL polypropylene microcentrifuge tubes. Forty microliters of CuSO_4_ (3.05 μg/mL) and 24 µL of BLM-A5 (5 μg/mL) were added. The mixture was vortexed and then transferred into Ostro^TM^ 96-well plate wells. Protein precipitation was achieved by the addition of 900 µL ice-cold acetonitrile with 0.1% formic acid. In-well mixing was performed with vigorous pipette aspiration and subsequently pushed through the Ostro^TM^ sorbent at 60 psi N_2_ for 5 min using a positive pressure manifold. Finally, the eluates were filtered through 0.2 µm reverse cellulose filters, generating a protein-, phospholipid-, and particulate-free solution. Since bleomycin has been proven to be light-sensitive [24], measures have been taken during all sample preparation steps to avoid direct light exposure.

### Instrumental analysis

Two analytical methods were optimized for the quantitative analysis of BLM. The analysis of biological samples with more complex matrices and at lower concentration levels was performed on a UHPLC-MS/MS system, while a simpler method employing an HPLC system with DAD detection was used to analyze BLM content in injection solution (Bleomedac®) at higher concentration levels.

#### Ultra-high-performance liquid chromatography–tandem mass spectrometry

For the instrumental analysis of trace level BLM in complex samples, an ultra-high-performance liquid chromatograph (UHPLC, Shimadzu, Kyoto, Japan) coupled to a hybrid quadrupole-linear ion trap mass spectrometry analyzer QTRAP 4500 (Sciex, Framingham, MA, USA) with positive electrospray ionization (ESI+) was utilized. Acquity^TM^ UPLC BEH Amide Column (1.7 µm, 2.1 mm × 50 mm) (Waters, Milford, MA, USA) column was used for separation. Separation was achieved using the gradient method and the following mobile phase composition: acetonitrile (mobile phase A) and 10 mM ammonium formate with 0.1% formic acid (mobile phase B). The gradient program started with 5% B, first increased to 50% at 2 min, and then further to 60% at 4 min. It then decreased back to 5% where it remained until 6 min. The total mobile phase flow rate was 0.3 mL/min. The column temperature was kept at 40 °C. An injection volume was 1.0 µL.

The ion source parameters were maintained as follows: ion spray voltage +5500 V; source temperature 650 °C; declustering potential (DP) 126 V; curtain gas (CUR) 40 psi; ion source gas 1 (GS1) 40 psi; ion source gas 2 (GS2) 25 psi. Ions were acquired in multiple reaction monitoring (MRM) mode; the transitions are together with the compound-specific parameters presented in Table 1.

**Table 1:** MRM parameters for the LC-MS/MS method

Compound	Q1 mass	Q3 mass	Time (ms)	CE (V)	CXP (V)
BLM-A2-Cu	738.4	707.2	500.0	23.0	4.0
BLM-B2-Cu	743.8	707.3	200.0	39.0	10.0
BLM-A5-Cu	752.3	715.7	200.0	37.0	8.0
BLM-A5-Cu	752.3	294.9	100.0	71.0	12.0

#### High-performance liquid chromatography

To perform this analysis, a high-performance liquid chromatograph coupled with a diode array detector (1260 Infinity Agilent Technologies, Santa Clara, CA, USA) was used. The separation was achieved using the Zorbax Eclipse C-18 column (150 mm × 4.6 mm, 5 µm) (Agilent Technologies, Santa Clara, CA, USA), which was kept at 40 °C. The injection volume was 10 μL. Gradient elution was performed with the following mobile phases: (A) 0.1% HCOOH in acetonitrile (mobile phase A) and 0.1% HCOOH in 20 mM NH4COOH formate buffer (mobile phase B). The gradient started with 95% B, which decreased down to 55% from minute 1 to 6 min; then, it increased back to 95% B at 6.5 min and was kept so until 9 min. The flow rate was 1 mL/min and the detector wavelength was set to 291 nm.

### Validation protocols

To confirm the *selectivity* of the method, blank and zero samples were evaluated. The blanks were prepared in appropriate matrices (serum or plasma) with the addition of internal standard but were not spiked with the analyte. The zero samples were prepared identically, though without the internal standard. The EMA criteria [21] for method validation require analyte peak areas in blanks to be ≤ 20% of the mean LLOQ analyte peak area and IS peak areas of the blanks below 5% of the average IS peak area of the calibrators and QCs.

*Carryover* was examined by analyzing solvent blanks after the measurement of the highest calibrator (1500 ng/mL). The blank analyte peak area, observed in the blanks should, according to the guidelines, not exceed 20% of the analyte peak area in the LLOQ. Likewise, the blank IS peak area should not exceed 5% of the mean IS peak area of the calibration standards and QC.

*Linearity* of the LC-MS was determined by analyzing matrix-matched calibration curves, made up of ten serum samples, spiked at different concentration levels in the range of 2.5–250 ng/mL in the final extract, corresponding to concentrations of 15–1500 ng/mL in serum as might be expected in real samples. For the HPLC method, the calibration curve consisted of six spiked samples, covering a concentration range of 2.5–40 µg/mL. The correlation between peak area ratio and bleomycin concentration was characterized by linear regression. EMA guidelines [21] consider a calibration to be acceptable if at least 75% of the calibrators fall within ± 15% (or 20% for the LLOQ) of their nominal concentrations.

*Sensitivity* of the method is defined in the FDA guidelines [25] as the lowest analyte concentration in the matrix that can be measured with acceptable accuracy and precision described by LLOQ. The determined LLOQ concentration was evaluated by analyzing three replicates of LLOQ concentration with each calibration curve, the mean of which should be within ±20% of the nominal concentration for at least 50% of the replicates.

*Accuracy*, described by trueness and precision, was evaluated by analyzing QC standards for three replicates at two different concentration levels. Precision reflected inter- and intra-day repeatability, the latter was evaluated by assessing injection and method repeatability. Injection repeatability was assessed as part of precision determination by successively injecting the same matrix-matched calibration standard three times while the method repeatability test included analyses of identically prepared (QC) samples at two concentrations, each in 3 replicates.

Inter-day precision and accuracy were part of the experiment following the method’s performance over a longer period of time. This was done by establishing a control chart, and recording quantification results for the quality control samples at two different concentrations, at a low (method LLOQ) and an intermediate concentration of the analyte, both in triplicates. Acceptance criteria, according to EMA guidelines [21] were (1) accuracy: mean concentration of QC samples must be within 15% of their nominal concentration (20% for QC at LLOQ). (2) Precision: each QC level should have a coefficient of variation (% CV) of no more than 15% and at the LLOQ no more than 20%.

*Extraction recovery* was calculated as an IS normalized response ratio of human serum spiked at LLOQ level and at 600 ng/mL before vs after extraction. The internal standard was added before the analysis in both cases to cover for instrumental drift.

#### Stability in serum and plasma

*Long-term stability:* eight 200 μL replicates of each, blank human plasma and serum, were spiked with BLM, generating concentrations of 1200 ng/mL. Duplicates were evaluated against freshly prepared matrix-matched calibration standards at four different time points: at 0, after 52, 90, and 123 days stored at −20 °C, protected from light.

*Freeze-thaw stability*: bleomycin freeze-thaw stability was evaluated for a total of six freeze/thaw cycles, from −20 °C to room temperature. Spiked blank serum and plasma samples were subjected to six cycles. The samples were compared against freshly prepared calibration standards.

#### Stability in injection solution

Long-term stability: bleomycin injection solution, obtained by dissolving the contents of one vial of Bleomedac® powder with 15,000 IU bleomycin in 5 mL of saline, was divided into aliquots; eight were stored in a freezer at −20 °C and another eight in a cooler at 8 °C. Duplicates were diluted (100 times; generating samples with 30 IU of BLM per mL; referring to biological potency) and analyzed against freshly prepared calibration standards after 0, 58, 98, and 141 days.

*Freeze-thaw stability*: injection solution with bleomycin was evaluated for six freeze/thaw cycles, prepared in the same way as plasma and serum.

*Measurement uncertainty* (*MU*) was estimated for both methods based on QC samples at two different analyte concentrations, which were acquired at different time points. MU took into account accuracy errors as well as the reproducibility of QCs. This covered uncertainty contributed by different analysts, days of analysis, and uncertainty in concentration contributed by the preparation of standards and calibration samples. An extended description of the calculation is reported in the Supplementary material.

*Traceability*: due to the lack of existing certified reference materials, otherwise necessary to demonstrate traceability, samples, prepared with BLM standards from two different producers (Cayman chemical and Santa Cruz Biotechnology), were compared. They were prepared at 600 ng/mL in three parallels for each.

## Results and discussion

### LC-MS-based method

#### Optimization

##### Instrumental analysis

The UHPLC-MS/MS method was essentially based on our previous method [11], which we transferred from an HRMS to a more sensitive and more robust MSMS system, a QTRAP MS, which is also more widely available in the clinical settings and generic analytical laboratories. The separation conditions were HILIC-like using the BEH Amide column. Several mobile phases and gradients were tested throughout the optimization process, but they all generated poorer peak shapes, hence the decision to keep ammonium formate buffer (10 mM NH_4_COO with 0.1% v/v formic acid, pH 3.3) and acetonitrile. A range of injection volumes was tested, determining 1 µL to fit best for the applied analyte concentration range. An increase to 10 µL might however be preferable for concentrations at the lower end of the applied concentration range. An overview of the tested and selected instrumental operational parameters is presented in Table S - 1.

##### Extraction and clean-up protocols

**Fig. 2:**
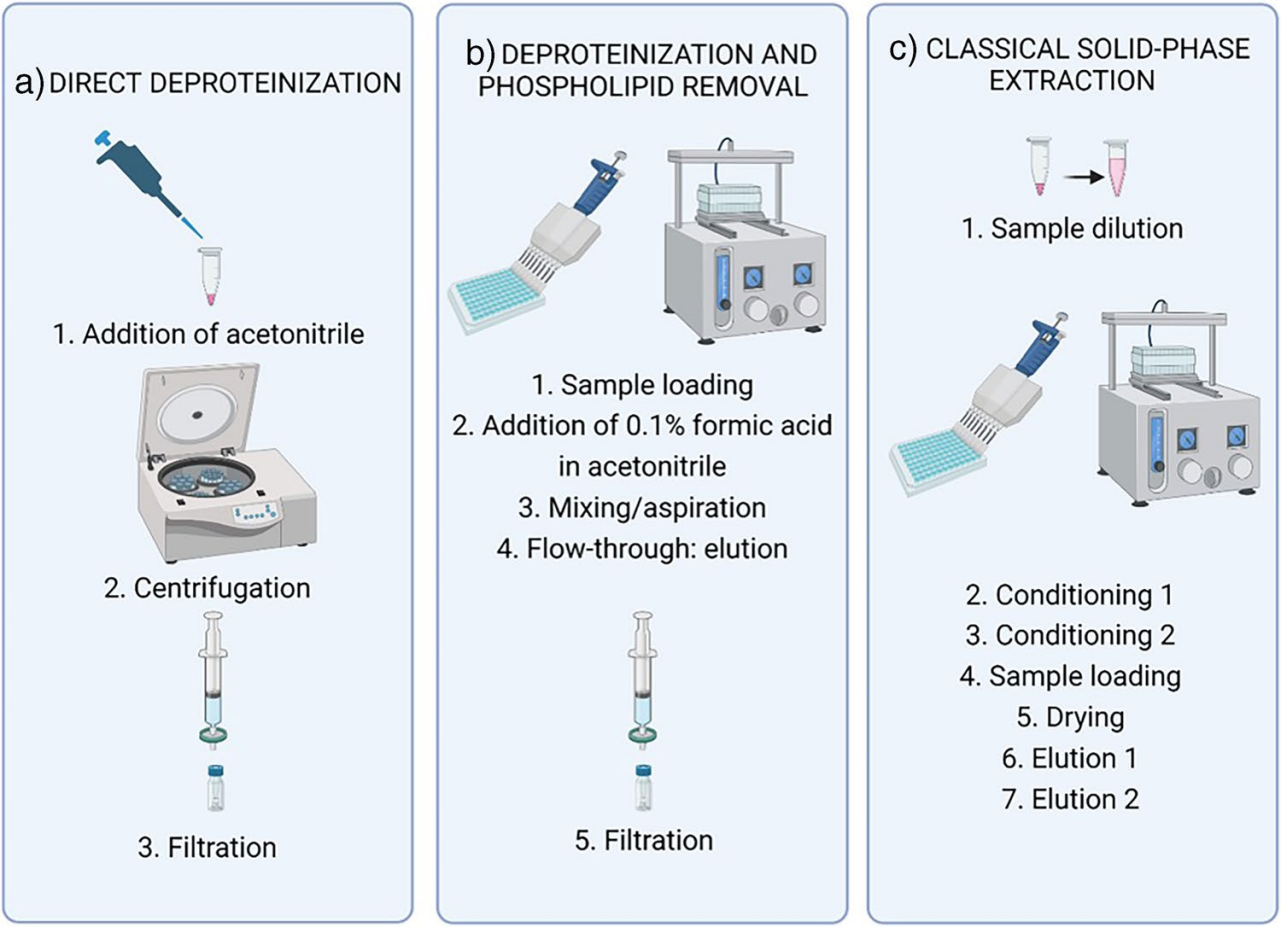
Sample preparation workflows

The existing sample preparation procedures developed for the clean-up of biological samples are time-consuming and laborious. Our main objective was therefore to develop a simplified protocol, which would facilitate the preparation of larger sample batches while still providing satisfactory clean-up and high recoveries. Three different procedures were compared based on their performance and overall complexity; the respective workflows are summarized in Fig. 2.

The simplest workflow (a), solely focused on removing protein, entailed straightforward acetonitrile-mediated protein precipitation, followed by centrifugation. An alternative method (b) provided the removal of protein as well as phospholipids, together comprising the main matrix interferences leading to ion suppression in MS analysis. Performed on a 96-well plate system, in-well deproteinization, achieved by forcefully adding ice-cold acetonitrile, was combined with subsequent removal of phospholipids through adsorption on C18 sorbent. The third method (c), classical SPE, was also performed on 96-well plate systems at positive N2 pressure. The universal polymeric HLB sorbent provided the removal of a wide range of matrix impurities.

The SPE method was originally considered to be the method of choice due to its ability to remove the largest spectrum of impurities in comparison to the other applied methods, including nonvolatile salts, which interfere with the MS analysis. The SPE protocol used hydrophilic-lipophilic-balanced (HLB) sorbent, packed into cartridges [11], and was within this study upgraded to a 96-well plate format, allowing preparation of a larger number of samples simultaneously, which drastically simplifies and speeds up the sample prep procedure. The SPE protocol was further optimized by modifying the composition of elution solvents. Different compositions of Milli-Q water, methanol, and acetonitrile were tested to determine, which provides the best results in terms of chromatographic peak shapes and recoveries (maximum elution efficiency and reduction in matrix effect). In HILIC chromatography, the peak shape is drastically affected by the choice of a reconstitution solvent. Yet, it was observed that the elution solvent cannot be completely reconstituted, since drying of the entire solvent leads to extensive losses of BLM as reported by Kosjek et al. [11], which is likely the consequence of its sorption to glass surfaces. It is therefore obvious that the resulting sub-optimal composition of elution solvent in turn affects the peak shape and chromatographic separation. Therefore, we aimed for the elution phase to contain as much ACN solvent as possible, limiting the MQ content in the final extract to a maximum of 30% in order to get acceptable peak shapes. However, since ACN performs rather poorly as an elution solvent (with both methanol and Milli-Q water eluting BLM at much higher efficiencies), the choice of the elution solvent composition was a compromise between the two factors, the optimal being methanol/Milli-Q water 1:1 (0.5 mL) followed by acetonitrile (0.5 mL). The comparison of all tested elution solvents (Table S - 2) as well as the final SPE protocol is presented in Supplementary material 2.2.1.

As an easier and faster alternative to initially employed SPE, the use of Ostro plates was considered, offering a combination of in-well protein precipitation with acetonitrile and subsequent phospholipid removal. The process, also performed on 96-well plates, is in this case advantageous as it is simpler and more rapid than the SPE. We followed the producer-recommended protocol, only implementing one slight modification. The concentration of formic acid added to the precipitating agent acetonitrile was reduced to 0.1% instead of 1% to avoid its negative impact on chromatographic peak shape.

Simplifying the pretreatment further by eliminating the extraction step an even more straightforward procedure was tested. Cold acetonitrile was added to the spiked serum or plasma, precipitating the protein, which was followed by centrifugation and finally filtering. The great repeatability as well as high recoveries (see Fig. S - 1) exhibited by this method would make it a preferred choice for serum samples. In the more complex plasma matrix, however, this is not the case as sufficient clean-up is not provided. More background noise in the chromatograms resulted in unfavorably high LLOQ. Additionally, the remainder of the matrix that is not removed causes signal suppression with the average response in spiked serum samples being over 30% lower than in serum samples at the same BLM concentration (see Table S - 3). Additionally, somewhat poorer repeatability is observed at low concentrations.

Figure S - 1 summarizes the performance parameters of the three described sample preparation methods (SPE, deproteinization with phospholipid removal on Ostro® plates, and direct deproteinization). All three sample prep protocols provided sufficient clean-up for serum. For plasma, however, the direct deproteinization method proved unsuitable, suggesting extraction step be necessary. The direct deproteinization method did however provide the highest recoveries, while these were not significantly different among the other methods. The variation coefficients are the lowest for the SPE method. Factoring in the simplicity and brevity of the procedure, the sample preparation method of choice was the one using phospholipid removal plates.

##### Further considerations

*Vial material*: possible effect of analyte sorption onto the surface of glass vials on quantitative results was studied by comparing glass and polypropylene plastic vials. Having observed no significant quantitative differences between the two, we assumed the container surfaces do not impact analysis.

*Addition of CuSO*_*4*_ (*agent for control of metal complex formation*): copper is ubiquitously present in the human body. As BLM has been shown to instantly form equimolar complexes in contact with copper ions [26] and the formation of such chelates in vivo is therefore practically inevitable in vivo, we determined BLM as well as the internal standard (BLM-A5) as BLM-copper complexes. The total copper content in blood plasma and serum is about 1000 ng/mL (15.7 nmol/mL) [27]. A large fraction thereof is bound to various protein species, either specifically (ceruloplasmin) or non-specifically (e.g., albumin). In sick individuals, the equilibrium between the bound and non-bound fractions can be altered [27]. To make sure that there were enough copper (Cu^2+^) ions available for the complexation of BLM, a surplus of CuSO_4_ (0.05µmol/sample) was added as an agent for control of metal complex formation. The samples with the addition of copper revealed about 18% higher abundancies of the analyte in comparison to those where Cu^2+^ was not added (see Fig. S - 2), indicating the necessity of this addition.

*Importance of the anticoagulant choice for plasma preparation*: we found the choice of blood collection tubes in sample withdrawal crucially important for the further analysis of the BLM-Cu complex. The most commonly used anticoagulant in plasma preparation is K2EDTA or K3EDTA, typically packed in purple-cap blood collection tubes for clinical hematology. A possible alternative is green-cap collection tube containing lithium heparin as an anticoagulant for plasma preparation, whereas a yellow-cap tube containing a clot activator and gel is used for serum separation. EDTA being a known chelating agent potentially affects quantification results by reducing the availability of free copper to BLM and in turn preventing the complexation of the entire BLM present. To assess whether this is of significance, plasma prepared with purple-cap and green-cap tubes was compared. Spiked samples were prepared at 600 ng/mL in triplicates for each plasma type. The clear difference between the average responses, being 88% lower for EDTA plasma, favors the use of heparin plasma (see Fig. S - 2).

*Implementation of a new internal standard*: the importance of an internal standard to normalize for potential errors and drifts that arise during sample preparation and LC-MS analysis, and to correct for matrix effects in MS detection, is indisputable. An isotopically labeled form of BLM would present the optimum choice since it mimics the behavior of the analyte, but since it is not available at the market, several alternative compounds were tested as potential internal standards, including methotrexate, vancomycin, and epirubicin [11]. Unfortunately, none of them provided a correction to linearity or reproducibility. Hence, the optimal alternative seems to be using any of its fractions, absent (or present in negligible quantities) in the reference standard and the clinically administered mixture. The A5 fraction was chosen for this purpose due to its commercial availability in a relatively pure form. We monitored its copper complex (BLM-A5-Cu). The exact masses of its protonated form and its characteristic fragments were first determined with a high-resolution mass spectrometer (quadrupole-time-of-flight MS) and then transferred to a low-resolution MS/MS setting. The MRM transitions are stated in Table 1. BLM-A5 was added to the samples at the concentration of 600 ng/mL and proved to provide an improvement in the method performance as seen in improved linearity (*r*^2^=0.9997 *vs r*^2^=0.9926 without normalization) and precision (RSD = 3.4% *vs* RSD = 16.1% without normalization for three replicates).

#### LC-MS method validation

To achieve reliable results, accurately reflecting concentrations of the analyte in the samples, the performance of the method was carefully evaluated. Validation was performed according to the EMA [21] guidelines, evaluating its selectivity, linearity, LLOQ, precision, accuracy error, and measurement uncertainty. Ascertained by using the exact BLM-A2-Cu and BLM-B2-Cu mass data, reported by Kosjek et al. [11] and by precisely determining the mass of newly introduced BLM-A5-Cu in the same way, the method exhibits good selectivity. The mass spectrum of the BLM-A5-Cu complex is presented in Fig. S - 4. Selectivity was then demonstrated by showing the response in blank samples, which were all well below the set limits (20% of the mean LLOQ analyte peak area for the analyte or 5% of the average IS peak area for the internal standard).

Quantification was based on the more abundant BLM-A2-Cu complex taking into account that A2 and B2 fractions are present in the same ratio in both reference standard and clinically used bleomycin. The exhibited response was linear in the whole analytical range (15–1500 ng/mL) with *r*^2^ values exceeding 0.99 for each calibration performed. LLOQ determined as the lowest calibration point with an accuracy error ≤20% was established at 15.0 ng/mL. The method proved to be accurate and repeatable, exhibiting errors within 10% of the nominal value for over 75% of the standards at each performed calibration. Method repeatability, assessed by measuring three replicates of samples at method LLOQ (15 ng/mL) as well as high (600 ng/mL) concentration, exhibited values up to 18.9% RSD and 16.8% RSD, respectively. The results are presented in Table S - 4.

Calibration samples were prepared in a serum matrix and were applicable for quantification in both serum and plasma samples, proving beforehand the matrix effect does not differ among the two in a way that would significantly impact the responses/results (Fig. S - 3). Two-sample *t*-tests assuming equal variances were performed at two concentration levels (15 and 600 ng/mL), comparing results obtained in both matrices and proving the absence of any significant differences. Some day-to-day variability in the slopes and intercepts, observed in the calibration curves, however, necessitates preparation and measuring of fresh calibration standards along with every sample batch.

##### Inter-day precision in accuracy: long-term performance of the analytical method

**Fig. 3:**
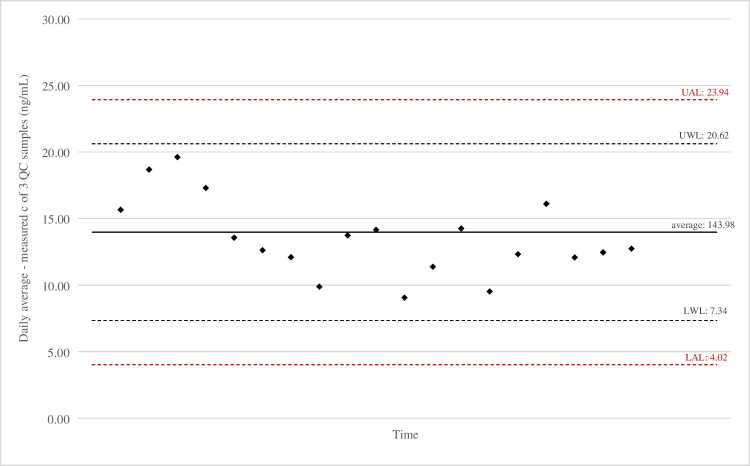
Control chart of quality control samples at low (15 ng/mL) concentrations of bleomycin

**Fig. 4:**
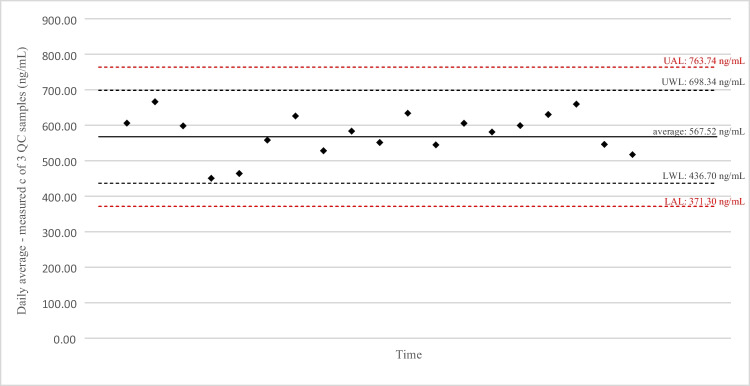
Control chart of quality control samples at medium (600 ng/mL) concentrations of bleomycin

Monitoring method performance over a longer time period was conducted by preparing multiple fresh calibration curves and quality control samples at different time points (approximately monthly intervals) by different analysts. The data, obtained from the QC samples, is displayed in form of a control chart for the LLOQ (15 ng/mL, Fig. 3) and high (600 ng/mL, Fig. 4) concentrations. The limits were calculated from the first 12 measured results, obtained within the first year, and were kept so for the reported time period. They are to be recalculated on an annual basis. The central line represents the average of all measurements as well as the upper and lower warning (UWL, LWL) and action (UAL, LAL) limits. These were established using the formulas:

$$\begin{array}{c}WL=\overline{x}\pm 2\cdot STDEV\\ AL=\overline{x}\pm 3\cdot STDEV\end{array}$$

where $\overline{x}$ stands for the average value of all the measurements and STDEV is the standard deviation between the daily averages, all applying to results, obtained at the first 12 measurements. Plotted on the charts are the average values, measured on each day along with the established limits, showing the scatter and accuracy of the results. As seen on the chart, values fall within said control limits, reflecting good method performance over the time period of 17 months. Variations in instrument signal strength and the resulting peak area variability across individual measurement days are reported in the Supplementary material Figure S - 5.

Dispersion of obtained measurement results can be explained with measurement uncertainty, an important parameter of the performance of an analytical method. To increase the confidence in produced results, evaluation of measurement uncertainty should therefore also be considered as a part of analytical validation. That is performed either by identifying individual uncertainty sources and evaluating the contribution of each one or, as a simpler alternative, by calculating it from validation data as described in guide ISO 21748 [28–31]. Once the uncertainty has been evaluated for a method in a particular laboratory, it may be applied to subsequently acquired results (if there is relevant QC data provided) [28].

The extended measurement uncertainty was calculated for two different concentration levels of bleomycin by following the Nordtest method [32]. The calculation is described in detail in Supplementary material 2.5.2. It was estimated to be 26% at high (600 ng/mL) and 50% at low (15 ng/mL) concentration of analyte at a 95% confidence interval.

##### Traceability

Among the validation parameters, th trueness of the measurements is the most challenging to demonstrate. It should ideally be assessed by relating the values to (internationally recognized) reference standards through an unbroken chain of calibrations with stated uncertainties, which describes traceability, a fundamental concept in metrology. Matching the real samples in matrix, concentration, and chemical form of the analyte, certified reference materials (CRMs) are considered to provide the highest level of accuracy, uncertainty, and traceability to an SI unit of measurement. Using such materials as quality control samples in method evaluation is the most straightforward way of establishing traceability. In case the market does not supply CRMs for a specific analyte/matrix, true traceability is impossible to achieve. Other measures can and should however be considered to demonstrate the accuracy of the results and increase confidence in them. One possibility is introducing external testing in the form of inter-laboratory studies provided there exist laboratories, performing analyses on the same type of samples. If this is not the case, other solutions, performed within one laboratory, must be resorted to.

Our approach was to compare the primary standard, used for the preparation of calibration samples, to a standard from a different producer. Results, shown in Fig. S - 6, show there is no significant difference between the two standards, which demonstrated a certain level of confidence in the quantification of results, at least regarding the adequacy of the applied primary standard.

### Bleomycin stability study

Information on the stability of the analyte in all relevant matrices, concentrations, and storage conditions is of fundamental importance to analytical science as it is the basis for determining the appropriate handling of samples and standards. When stability information for the relevant conditions is not previously reported, a stability study should be conducted along with bioanalytical method validation. The results of quantitative analyses which are seldom performed straight after sample collection can only accurately reflect analyte concentrations at sampling time if it has not degraded in the meantime. No less important is the data on analyte stability in standard solutions used for method calibration and quality control. There is limited information available regarding the stability of bleomycin; what does exist focuses mostly on its photolability and the consequent need for protection from exposure to light [24]. Hence, data on its long-term stability in biological matrices will be of added value for more accurate guidance in clinical workflows.

Bleomycin stability was investigated for biological matrices (plasma and serum) and injection solution at storage-relevant conditions. Data on its stability in biological samples (long-term freezer stability and freeze-thaw stability) provided useful information for sample handling from collection to analysis whereas the data for its long-term freezer and cooler stability in the physiological solution provided information useful for creating/establishing storage recommendations for prepared injection solutions.

Recoveries of the measurements for long-term and freeze-thaw stability experiments are displayed in Figs. S - 7 and 8, each point presenting the average recovery of two aliquots. Results for all measurements were within ± 14.0% of the initial value. Considering the calculated extended measurement uncertainty of the method at the relevant concentration range is higher than this percentage (26.2% at high 600 ng/mL), these results suggest bleomycin remains stable in the two matrices under the applied conditions for the investigated time.

The stability of BLM in injection solution was investigated to explore the possibility of preparing BLM solutions in advance and storing them before use. The results shown in Fig. S - 9 indicate that BLM remains stable over the given time period both when stored in a cooler or in a freezer with the results varying no more than 5% within the period of 140 days. In Fig. S - 10, the results of a freeze-thaw experiment are presented. The measured values indicate its stability throughout the six freeze-thaw cycles, the deviations from the initial value all falling well within the calculated measurement uncertainty of the method.

## Conclusions

Through an example of the development, optimization, and establishment of quality assurance protocols for two analytical methods, this study describes a quantitative analysis of a complex polar analyte on the example of bleomycin, addressing several of the challenges that can be faced in such analyses. For the quantification of BLM in blood-derived biological samples, an existing LC-MS method [11] was taken as the basis and was improved by modifying the sample preparation procedure as well as transferring the instrumental part to a more sensitive and accessible instrument. The final method comprises a considerably simpler and more time-efficient sample preparation protocol and analysis on a UHPLC-MS/MS system. Efficient and accurate analysis of large sample sizes is essential in routine clinical practice. Our method provides a simple, sensitive, and reliable approach that can produce high-quality results, making it ideal for patient monitoring, established therapy regimes, and potential therapy optimization studies. With the possibility of mitigating the severe side effects and improving efficacy through combination with other drugs or therapies, bleomycin treatment warrants further investigation, and our method offers a promising platform for such studies.

For the purpose of quantifying BLM in its clinically used form, an injection solution that represents an entirely different sample type, a simple HPLC-based method, was developed and validated. Separation of BLM’s main fractions was, unlike in many previously published methods, achieved in a rapid manner and without the addition of ion-pairing reagents. Full validation, performed for both methods, included assessment of measurement uncertainties and demonstration of the analyte stability.

This work supports enhanced integration of metrological concepts in analytical chemical methods to assure good quality and control of measurement results, highlighting the utmost importance of this, especially when dealing with challenging and complex analyses. It considers the issues of traceability and supports the applicability of measurement uncertainty as well as other important metrological concepts that are most often underreported in scientific articles. This study is also an explanatory example of how the performance of any complex method should be, in addition to initial validation, controlled over time in order to constantly assure an acceptable quality of results. Data, acquired in regular QC checks are visually presented in the form of a control chart, allowing easy tracking of the accuracy and dispersion of QC sample results as well as any trends that might emerge.

While this method covers the analysis of blood-derived samples, its application could be extended to other biological samples with additional modifications to the sample preparation procedure and instrumental analysis. These include tissue samples with even more complex matrices such as cell culture samples, used in ECT studies. For the latter, special efforts should be directed towards achieving lower LLOQ limits indicating the need for (at least) a more sensitive instrument.

## Supplementary Information

Below is the link to the electronic supplementary material.

Supplementary file1 (DOCX 146 KB)

## Abbreviations

BLM: Bleomycin
LLOQ: Lower limit of quantification
BLM-A2-Cu: Bleomycin A2 fraction copper complex
BLM-B2-Cu: Bleomycin B2 fraction copper complex
ACN: Acetonitrile
ECT: Electrochemotherapy
BLMH: Bleomycin hydrolase
