# Supplementary material for: An effective validation of analytical method for determination of a polar complexing agent: the illustrative case of cytotoxic bleomycin
Source: Anal Bioanal Chem. 2023 Apr 12;415(14):2737–48. doi: 10.1007/s00216-023-04675-x (PMC10185589; doi:10.1007/s00216-023-04675-x)
Supplement: Supplementary file 1 — Supplementary file1 (DOCX 146 KB) [file 216_2023_4675_MOESM1_ESM.docx]

Electronic Supplementary Material

to the paper

**An effective validation of analytical method for determination of a polar complexing agent: the illustrative case of cytotoxic bleomycin**

Helena Plesnik^1,2^, Masa Bosnjak^3,4^, Maja Cemazar^3,5^, Gregor Sersa^3,6^, Tina Kosjek^1,2^*

^1^Department of Environmental Sciences, Jozef Stefan Institute, Jamova 39, 1000 Ljubljana, Slovenia

^2^International Postgraduate School Jozef Stefan, Jamova cesta 39, 1000 Ljubljana, Slovenia

^3^Department of Experimental Oncology, Institute of Oncology Ljubljana, Zaloska 2, 1000 Ljubljana, Slovenia

^4^Faculty of Pharmacy, University of Ljubljana, Askerceva 7, 1000 Ljubljana, Slovenia

^5^Faculty of Health Sciences, University of Primorska, Polje 42, 6310 Izola, Slovenia

^6^Faculty of Health Sciences, University of Ljubljana, Zdravstvena pot 5, 1000 Ljubljana, Slovenia

*Correspondence to: Tina Kosjek, E-mail: [tina.kosjek@ijs.si](mailto:tina.kosjek@ijs.si), Tel.: +38614773288

**Contents**

[2. Supplementary material to Results and Discussion 3](#_Toc130558175)

[*2.1 Optimization of LC-MS parameters* 3](#_Toc130558176)

[*2.2 Sample preparation procedure* 3](#_Toc130558177)

[*2.2.1 Optimization and comparison of alternative sample preparation protocols* 3](#_Toc130558178)

[*2.2.2 Other sample preparation modifications* 5](#_Toc130558179)

[*2.2.3 Implementation of a new internal standard* 6](#_Toc130558180)

[*2.3* *Signal strength quality control* 6](#_Toc130558181)

[*2.4 HPLC-based method development* 7](#_Toc130558182)

[*2.4.1 HPLC method validation* 8](#_Toc130558183)

[*2.5 Validation results* 8](#_Toc130558184)

[*2.5.1 Traceability* 8](#_Toc130558185)

[*2.5.2 Estimation of measurement uncertainty* 8](#_Toc130558186)

[*2.6 Stability study* 10](#_Toc130558187)

# Supplementary material to Results and Discussion

## *2.1 Optimization of LC-MS parameters*

Table S - 1: Tested and selected instrumental operational parameters

| **Tested parameter** | **Tested variables** | **Selected/optimal** |
| --- | --- | --- |
| **Organic mobile phase (A)** | Acetonitrile  Acetonitrile + 0.1% formic acid | Acetonitrile |
| **Injection volume** | 1-5 µL | 1 µL |
| **Interscan delay** | 100-500 ms | 500 ms (BLM-A2-Cu)  200 ms (BLM-B2-Cu)  200 ms (BLM-A5-Cu)  100 ms (BLM-A5-Cu) |
| **Resolution Q1 and Q2** | Unit, Low | Unit |

## *2.2 Sample preparation procedure*

### *2.2.1 Optimization and comparison of alternative sample preparation protocols*

Elution solvents for the first elution step of SPE have been tested in various ratios as illustrated in Table S-2. Highest recoveries were present for either MeOH or MeOH/MQ 1:1. The latter was chosen due to lower variation among the parallels.

Table S – 2: Elution from Oasis HLB^TM^ sorbent: Solvent optimization

| Elution solvents | | Average AUC 2 parallels (RSD %) |
| --- | --- | --- |
| Elution 1 (0.5 mL) | Elution 2 and 3 (2·0.5 mL) |  |
| MeOH | ACN | **686 500** (3.8 %) |
| MQ/MeOH 1:9 |  | 642 500 (5.1 %) |
| MQ/MeOH 1:4 |  | 575 500 (9.5 %) |
| MQ/MeOH 3:7 |  | 594 000 (2.4 %) |
| MQ/MeOH 1:1 |  | **687 000** (1.2 %) |
| MQ/MeOH 3:2 |  | 587 000 (6.8 %) |

*Final SPE protocol*

200 µL of matrix (plasma or serum) was put into 1.5 mL polypropylene microcentrifuge tubes, adding CuSO4 and spiking it with the analyte and internal standard. 0.1% formic acid in MQ was added to obtain a total volume of 1 ml. After vortexing, the mixtures were transferred to SPE 96-well plates (Oasis HLB 96-well plate, 30 mg), pre-conditioned with 1 mL of methanol and 0.1% formic acid in MQ, respectively. All solvents and samples were pushed through the sorbent with nitrogen on a positive pressure manifold. Drying was performed for 10 minutes at 15 psi N2. Elution consisted of three steps, 0.5 mL of MeOH/MQ followed by 0.5 mL ACN. The eluate was finally dried to 1 mL.

Direct deproteinization protocol

200 µL of matrix (plasma or serum) was put into 1.5 mL polypropylene microcentrifuge tubes, adding CuSO4 and spiking it with the analyte and internal standard. Cold acetonitrile was added to obtain a total volume of 1 ml. The mixture was vortexed and sonicated for 10 minutes, which was followed by centrifugation (20 minutes at 12900 RCF), and, finally, filtration through RC filters (0.2 µm).


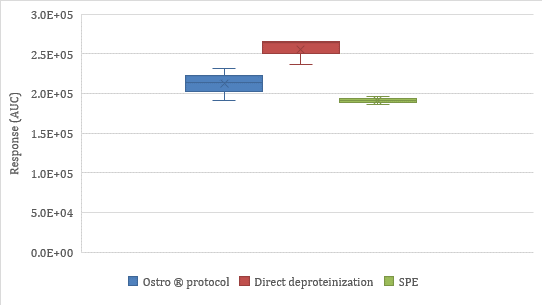


|  | AUC (RSD; n=3) |
| --- | --- |
| Ostro ® protocol | 212 000 (9.7%) |
| Direct deproteinization | 256 000 (6.4 %) |
| SPE | 191 000 (2.9 %) |

Figure S – 1: Comparison of signal intensities (AUC) among three sample preparation protocols (serum as matrix)

Table S – 3: Suitability of direct deproteinization method for plasma samples

|  | AUC (RSD; n=3) plasma | AUC (RSD; n=3) serum |
| --- | --- | --- |
| 5 ng/mL BLM | 7477 (25.2 %) | 10993 (10.0%) |
| 50 ng/mL BLM | 49767 (10.4 %) | 81867 (13.2 %) |

### *2.2.2 Other sample preparation modifications*


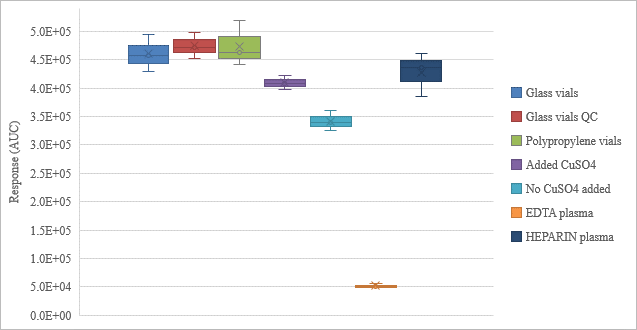


Figure S – 2: Role of glass material, addition of complex formation agent (CuSO4) and anticoagulant type for plasma collection


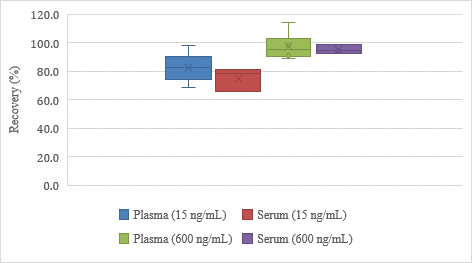


Figure S – 3: Comparison between serum and plasma matrix: Normalized response

### *2.2.3 Implementation of a new internal standard*

The exact mass and viable fragments of BLM-A5-Cu complex (to be used as internal standard) was precisely determined with an HRMS scan. The 2+ charged ions expressed higher intensity than 1+ charged, hence the use of the double charged BLM-A5-Cu with the measured m/z 752.272 as the parent ion.


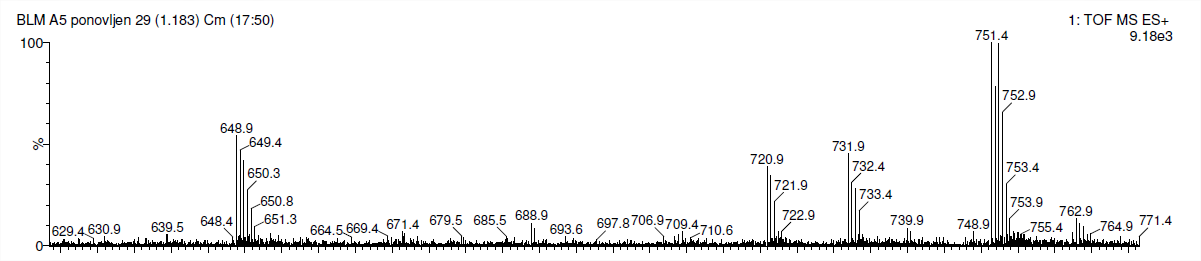


Figure S - 4: Mass spectrum of BLM-A5-Cu

## *Signal strength quality control*

Figure S – 5: Peak areas of QC samples at two concentrations through time, corresponding the measurements on the control charts (averages of three values)

## *2.4 HPLC-based method development*

An HPLC method was developed with the purpose of analyzing BLM in a higher concentration range and in simple matrices, i.e. for the study of BLM stability in injection solution. The challenge, often pointed out with methods using UV detection is achieving chromatographic separation of structurally very similar BLM-A2 and BLM-B2 fractions, which is not an issue when using MS detection.

Method development entailed testing a variety of different mobile phase compositions, including methanol and acetonitrile with and without the addition of formic acid as organic and ammonium formate buffer as the aqueous phase. The optimal mobile phases were determined to be 0.1% HCOOH in acetonitrile and buffer containing 20 mM ammonium formate with 0.1% HCOOH. The separation of A2 and B2 fractions was achieved without the addition of any ion-pairing reagents, which were before used in multiple published reversed-phase HPLC methods [12, 13, 18, 19] to aid the separation of individual fractions. Said fractions eluted at 4.7 and 5.0 minutes, respectively.

### *2.4.1 HPLC method validation*

Having successfully separated major BLM-A2 and BLM-B2 fractions, quantification was performed for the predominant BLM-A2 fraction. Method validation included evaluation of selectivity, linearity, LLOQ, precision, accuracy error, and measurement uncertainty. Good selectivity of the method was demonstrated by the absence of signals at critical retention times for all of the blank samples. The response throughout the analytical range (2.5-40 µg/mL) was proved linear with R^2^ values over 0.999 for all of the calibration curves prepared/measured. The results also showed negligible day-to-day variability in the slopes and intercepts. LLOQ determined as the lowest calibration point with accuracy error ≤20 % was established at 2.5 µg/mL (and 5.0 µg /mL for BLM-B2 fraction) and was found to be sufficiently low for the intended purpose. Individual calibration samples all exhibited recoveries deviating from the nominal value by less than 10% (97 - 103%) for the values above LLOQ and by 20 % (90-112 %) for samples at LLOQ. RSD values for replicates (at LLOQ and at 30 µg/mL) were all below 2.9 %, demonstrating sufficient method precision. Extended measurement uncertainty using the calculation described in Supplementary material was evaluated for this method as well and was determined to be 32.2% (for LLOQ at 2.5 µg/mL) or 9.3 % (at high concentrations; 30 µg/mL).

## *2.5 Validation results*

### *2.5.1 Traceability*


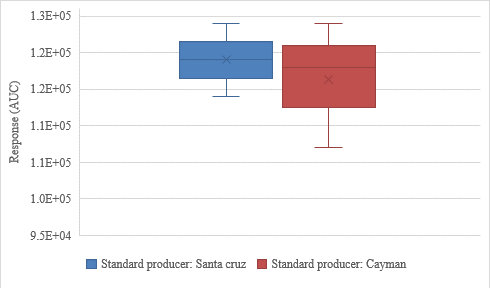


Figure S – 6: Comparison between primary standards from different producers: Santa Cruz and Cayman

### *2.5.2 Estimation of measurement uncertainty*

Combined measurement uncertainty was calculated from the reproducibility and bias of the QCs using the formula:

$$u\left( combined \right)=\sqrt{{u(reproducibility)}^{2}+{u(bias)}^{2}}$$

***u(reproducibility)*** is relative standard deviation (RSD) in QC samples measured within different batches on different days. QC samples took into account uncertainty contributed by sample preparation as well as the instrumental repeatability. Information about the latter was obtained by triple injection of the same QC sample. QC samples were prepared at two different concentrations by spiking serum with 12.5 and 600 ng/mL of standard.

***u(bias)*** was calculated with the formula:

$$\sqrt{{accuracy error}^{2}+({\frac{RSD(QC)}{\sqrt{N}})}^{2}+{u(concentration)}^{2}}$$

It takes into account the bias of accuracy samples, reflecting method bias, reproducibility of response for QC samples and uncertainty in concentration derived by the preparation of primary and working standards.

***Accuracy error*** represents difference between nominal and measured concentration for each concentration level. *RSD(QC)* is relative standard deviation among the daily averages of the QC samples, which was also used to define *u(reproducibility)*. We assumed that *RSD(QC)* is would not be different from that of the real samples.

***N*** represents the number of measurement events, each time three QCs were measured and averaged.

***u(concentration)*** is uncertainty in concentration of the calibration and QC samples. It takes into account uncertainty contributed from volume measurements (volumetric flasks and pipettes). Uncertainty in concentration was calculated in accordance with the rules of uncertainty propagation for multiplication relations, taking into account uncertainty contributions in each step of standard preparation:

$$u\left( concentration \right)=\sqrt{2{u(pipette)}^{2}+2{u(volumetric flask)}^{2}+{u(mass)}^{2}}$$

The uncertainty of the standard purity were not included in the calculation as the certificate of analysis for the used standard batch provided assay information (95.7%) without stated uncertainty. Uncertainty of the mass describes the uncertainty of the scale, defined per last calibration certificate.

Uncertainty in volume of volumetric flask is defined by the producer. Uncertainty in volume measured by pipette was calculated from data obtained in monthly check of pipettes conducted in our laboratory, performed by weighing certain volumes of calibration liquid (water) and taking into account the correction for density according to temperature and pressure at the time of measurement.

*Table S - 4: Uncertainty parameters (LCMS method)*

|  | *accuracy error (%)* | *method repeatability (RSD %)* | *instrumental repeatability (RSD %)* | *reproducibility (RSD %)* | *u(combined) %* | *u(extended) %* |
| --- | --- | --- | --- | --- | --- | --- |
| *c=15 ng/mL* | *10.5%* | *8.7%* | *1.8%* | *21.9%* | *25.0%* | *49.9%* |
| *c=600 ng/mL* | *4.2%* | *8.1%* | *1.3%* | *10.6%* | *13.1%* | *26.2%* |

*Table S - 5: Uncertainty parameters (HPLC method)*

|  | *accuracy error (%)* | *method repeatability (RSD %)* | *instrumental repeatability (RSD %)* | *reproducibility (RSD %)* | *u(combined) %* | *u(extended) %* |
| --- | --- | --- | --- | --- | --- | --- |
| *c=2.5 µg/mL* | *9.5%* | *2.4%* | *0.22%* | *12.8%* | 16.1% | *32.2%* |
| *c=30 µg/mL* | *2.2%* | *0.5%* | *0.3%* | *3.3%* | 4.7% | *9.3%* |

## *2.6 Stability study*

Figure S - 7: Long-term stability of bleomycin in plasma and serum

Figure S - 8: Freeze-thaw stability of bleomycin in plasma and serum

Figure S - 9: Long-term stability of bleomycin in injection solution

Figure S - 10: Freeze-thaw stability of bleomycin in injection solution
